# Exploration of semen quality analyzed by casa-mot systems of brahman bulls infected with BLV and BHV-1

**DOI:** 10.1038/s41598-023-45981-9

**Published:** 2023-10-31

**Authors:** Derling Pichardo-Matamoros, Francisco Sevilla, Jorge Elizondo-Salazar, Carlos Jiménez-Sánchez, Eduardo R. S. Roldan, Carles Soler, Sabrina Gacem, Anthony Valverde

**Affiliations:** 1 National Agrarian University, Juigalpa Campus, Chontales, 55000 Nicaragua; 2 Graduate Program in Agricultural Sciences and Natural Resources, University of Costa Rica, 11501 San Pedro de Montes de Oca, Costa Rica; 3 Animal Reproduction Laboratory, School of Agronomy, Costa Rica Institute of Technology, San Carlos Campus, Alajuela, 223-21002 Costa Rica; 4 Faculty of Agri-Food Sciences, Alfredo Volio Mata Experimental Station, University of Costa Rica, 30304 Cartago, Costa Rica; 5 Tropical Diseases Research Program, Faculty of Health Sciences, Veterinary Medicine School, National University of Costa Rica, Benjamín Núñez Campus, 40101 Barreal de Heredia, Costa Rica; 6 Department of Biodiversity and Evolutionary Biology, National Museum of Natural Sciences, Spanish National Research Council (CSIC), 28006 Madrid, Spain; 7 Department of Cellular Biology, Functional Biology and Physical Anthropology, University of Valencia, Campus Burjassot, C/Dr Moliner, 50, 46100 Valencia, Spain; 8 Department of Animal Medicine and Surgery, Universitat Autònoma de Barcelona, Campus UAB, Edifici V, Bellaterra (Cerdanyola del Vallès), 08193 Barcelona, Spain

**Keywords:** Animal biotechnology, Animal physiology

## Abstract

Enzootic bovine leukosis virus (BLV) and bovine herpesvirus 1 (BHV-1) are very important infectious agents for the livestock industry worldwide. The present study aimed to explore the association between natural exposure to BLV and BHV-1 with sperm quality analyzed by Computer-Assisted Semen Analysis (CASA) systems. Ten sexually mature Brahman bulls, with sanitary status BLV^+^/BHV-1^+^ (n = 2), BLV^−^/BHV-1^+^ (n = 6) and BLV^-^/BHV-1^-^ (n = 2) were evaluated twice, 30 days apart. Results showed that sanitary status of each bull was not associated with semen quality. It was found that the quality of the semen from the second collection was better due to the interruption of sexual rest. The evidence thus revealed that a bull infected with BLV generated good-quality contaminated semen and, therefore, that it is essential to detect contaminated seminal samples to prevent the spread of BLV. A multivariate analysis showed the presence of four sperm subpopulations in Brahman bulls that differ significantly in their kinematic patterns and with respect to sanitary status (P < 0.05), indicating that infection-free and seronegative bulls present the best kinematic parameters, which improved discrimination of sperm quality according to sanitary status. Overall, the analyses indicate that the seropositive-infected bulls with BLV and BHV-1 should be excluded from beef cattle farms.

## Introduction

Reproductive efficiency has an important impact on the profitability of the livestock industry. Reproduction is a very complex process which depends on the fertility qualities of the cow and the bull, with a significant percentage of reproductive failure attributable to bull subfertility^1^. Fertility potential of a bull is observed mainly through the analysis of sperm quality^1,2^. In extensive systems of beef cattle breeding, fertility evaluations revealed that only 20% of bulls are suitable for mating^2^. Consequently, the low availability of bulls with acceptable fertility is a problem and predicting it is complex^3^, even more when the number of infertile or sub-fertile bulls is related to infectious diseases during their productive life. These infections can cause temporal or permanent infertility problems.

Among the most common infectious agents associated with reproductive failure are bovine viral diarrhea virus (BVDV), bovine herpesvirus 1 (BHV-1), *Tritrichomonas*, *Neospora* and *Campylobacter*. Other important endemic agents are enzootic bovine leukosis virus (BLV), *Leptospira*, and *Brucella abortus*^2,4–6^. A single exposure to BHV-1 can have a high economic impact and commercial importance for artificial insemination (AI) centers due to impaired sperm quality or transmission risk to healthy bulls^2,7^. The same applies to BLV^5,8–10^. Both viruses, BLV and BHV-1, are frequently found in multiple infections in endemic regions^11,12^.

Depending on the animals' physiological conditions and environmental factors, simultaneous viral infections show interactions that can lead both to increased or reduced adverse effects on health status^13–15^. It is known that the association of BLV and BHV-1 have a negative effect on semen quality^4,7,9^, but their multiple effect on bull sperm function has not been elucidated. BHV-1 has been reported to cause infertility or reduced reproductive efficiency, although other authors found no significant association between simple exposure to the virus and seminal variables such as concentration, volume, mass and progressive motility^2^. Another study determined in bulls infected with BHV-1 an increase in sperm morphological abnormalities, decreased motility and swimming capacity, membrane integrity, and sperm concentration compared to non-infected bulls^7^, However, in both studies seminal parameters were assessed by subjective analyses and sperm quality has been just examined using optical microscopy observations.

Currently, a commercial computer-assisted semen analysis system (CASA) allows to determine with precision and objectivity parameters such as sperm swimming, kinematics, morphometrics sperm variables and sperm concentration to evaluate semen quality and to be able to predict bull fertility^16–18^. Therefore, the aim of this study was to explore the association between exposure to BLV and/or BHV-1 with semen quality determined by CASA technology.

## Results

In this study, blood samples showed that two bulls were seropositive and infected with BLV and BHV-1 (BLV^+^/BHV-1^+^), six bulls were seropositive and latently infected with BHV-1 (BLV^−^/BHV-1^+^), and two bulls were negative for the presence of antibodies and antigen associated with the BLV and BHV-1 (BLV^−^/BHV-1^−^). All bulls maintained the sanitary status throughout the investigation. Furthermore, only one seminal sample from bull 498 was positive to BLV at 0 day but it was negative at 30 days (second seminal sample). The BLV detected in blood and seminal samples of bull were characterized as genotype 5 of BLV (strain 370322-498BlSp-CR, GenBank accession number: OP718193 (https://www.ncbi.nlm.nih.gov/nuccore/OP718193; accessed on 17 July 2023). No blood and semen samples were positive for the presence of BHV-1.

**Figure 1:**
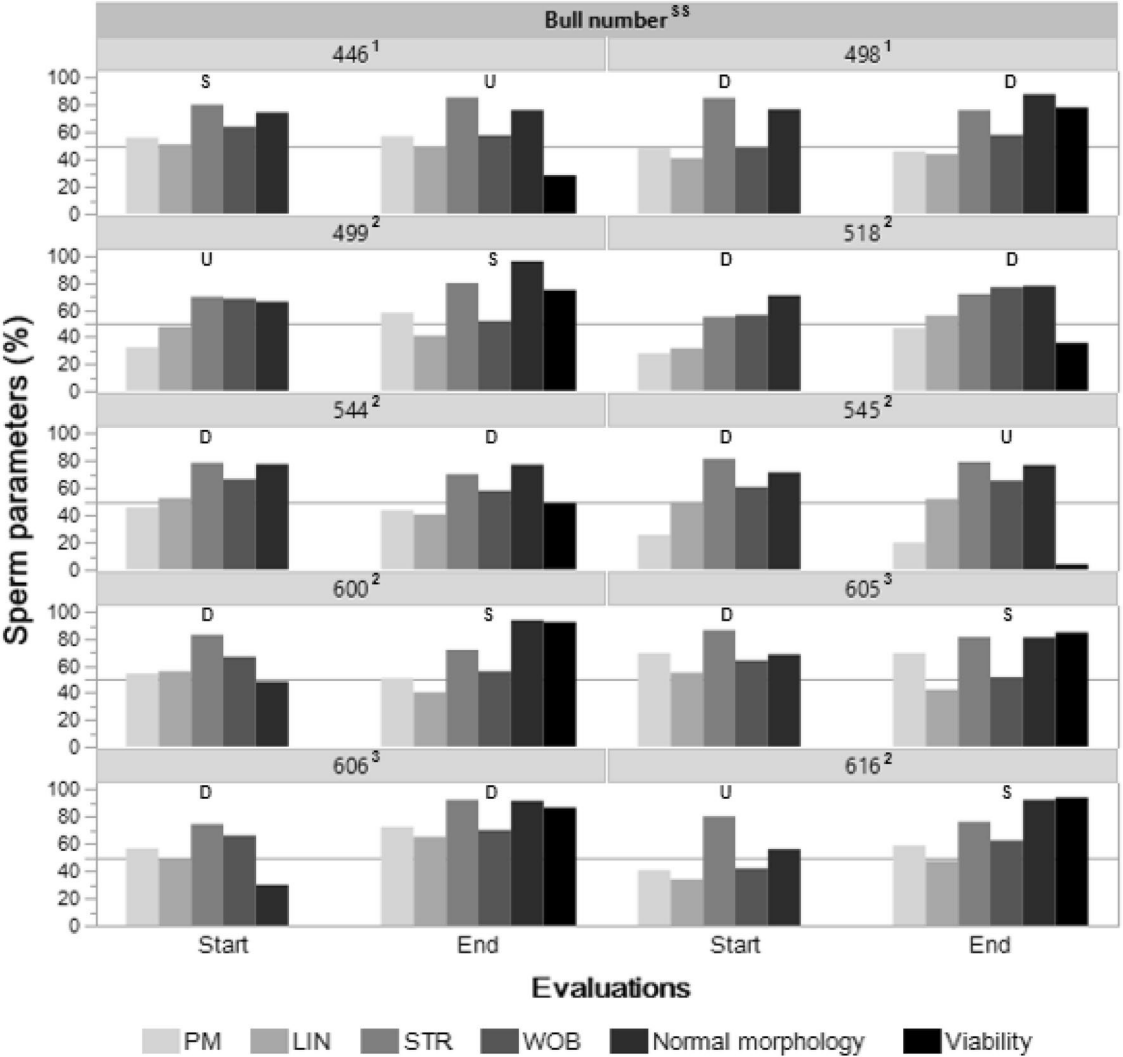
Changes of sperm parameters and sperm quality of bulls with different sanitary status. *SS* sanitary status: 1 = BLV^+^/BHV-1^+^, 2 = BLV^−^/BHV-1^+^, 3 = BLV^−^/BHV-1^−^. Sperm quality: S = satisfactory, D = doubtful, U = unsatisfactory. Sperm parameters: PM = progressive motility, LIN = linearity of forward progression, STR = straightness, WOB = wobble. The horizontal lines indicate values of 50%.

The volume per ejaculate ranged from 2 to 12 mL, whereas the mean concentration ranged from 388 to 1560 M/mL and the total motility ranged from 37.7 to 71.0%. Progressive motility (PM) and kinematics variables did not show a change in trend between evaluations (0 and 30 days) or between the sanitary status of the bulls that would allow differentiation or increase the discriminant potential on the seminal quality initially characterized based on the percentage of normal morphology and viability (Tables 1 and 2, Fig. 1).

**Table 1:** Sanitary status, means of sperm parameters and semen quality of Brahman bulls (n = 10) at the beginning of the study.

Parameters	Bull number									
	446^1^	498^1^	499^2^	518^2^	544^2^	545^2^	600^2^	605^3^	606^3^	616^2^
TM (%)	82.5	55.5	55.0	68.0	65.5	34.5	73.5	86.5	91.0	53.0
PM (%)	56.0	48.0	32.5	28.0	45.5	25.5	54.5	69.5	56.5	40.5
VCL (μm/s)	133.0	92.0	164.5	116.5	107.0	123.5	157.0	152.0	155.5	95.5
VSL (μm/s)	68.5	38.5	80.0	36.0	56.5	62.0	88.5	84.5	77.0	33.0
VAP (μm/s)	85.0	45.0	113.5	67.0	71.5	75.5	106.0	97.5	103.0	41.0
ALH (μm)	4.0	3.0	4.0	2.5	2.5	3.0	4.0	4.0	4.5	4.0
LIN (%)	51.0	41.0	48.0	31.5	52.5	50.0	56.0	55.0	49.0	34.0
STR (%)	80.0	85.0	70.0	55.0	78.5	81.5	83.0	86.5	74.5	80.0
WOB (%)	64.0	48.5	68.5	56.5	66.5	60.5	67.0	64.0	66.0	42.0
BCF (Hz)	10.0	12.0	8.5	10.5	11.5	12.5	9.5	13.0	10.5	10.0
Normal Morphology (%)	74.3	76.6	66.1	70.9	77.4	71.3	48.4	68.5	30	56.0
Semen quality	*S*	*D*	*U*	*D*	*D*	*D*	*D*	*D*	*D*	*U*

*PM* progressive motility, *TM* total motility, *VCL* curvilinear velocity, *VSL* straight-line velocity, *VAP* average path velocity, *ALH* amplitude of lateral head displacement, *LIN* linearity of forward progression, *STR* straightness, *WOB* wobble, *BCF* beat-cross frequency, *S* satisfactory, *D* doubtful, *U* unsatisfactory.

^1^Seropositive-infected bull with BLV and BHV-1.

^2^Seropositive-latently infected bull with BHV-1.

^3^Negative bull to BLV and BHV-1.

**Table 2:** Sanitary status, means of sperm parameters and semen quality of Brahman bulls (n = 10) at the end of the study (30 days).

Parameters	Bull number									
	446^1^	498^1^	499^2^	518^2^	544^2^	545^2^	600^2^	616,605^2^	605,606^3^	606,616^3^
TM (%)	75.0	74.0	83.0	74.5	76.0	35.0	85.0	92.7	93.5	80.5
PM (%)	57.0	46.0	58.0	47.0	43.5	20.0	51.0	58.7	69.5	72.5
VCL (μm/s)	166.0	169.0	178.0	144.5	146.5	100.5	174.0	180.5	137.5	86.0
VSL (μm/s)	83.0	76.0	74.0	81.5	59.0	52.5	71.0	86.7	58.0	56.0
VAP (μm/s)	96.0	99.0	93.0	112.0	84.5	66.0	98.0	113.2	71.0	60.5
ALH (μm)	5.0	4.0	5.0	3.0	4.0	3.0	4.5	5.0	4.0	2.0
LIN (%)	49.5	44.0	41.0	56.0	40.5	52.0	40.5	47.7	42.5	65.0
STR (%)	85.5	76.0	80.0	72.0	70.0	79.0	72.0	76.2	81.5	92.0
WOB (%)	57.5	58.0	52.0	77.0	58.0	65.5	56.0	62.5	51.5	70.0
BCF (Hz)	11.0	12.0	11.0	8.0	11.0	7.0	12.0	10.5	11.0	11.0
Normal Morphology (%)	76.2	87.7	96.2	78.0	77.0	76.6	93.8	92.0	81.2	91.3
Viability (%)	28.2	78.0	75.1	35.8	49.0	4.0	92.7	93.8	84.9	86.5
Semen quality	*U*	*D*	*S*	*D*	*D*	*U*	*S*	*S*	*S*	*D*

*PM* progressive motility, *TM* total motility, *VCL* curvilinear velocity, *VSL* straight-line velocity, *VAP* average path velocity, *ALH* amplitude of lateral head displacement, *LIN* linearity of forward progression, *STR* straightness, *WOB* wobble, *BCF* beat-cross frequency, *S* satisfactory, *D* doubtful; *U* unsatisfactory.

^1^Seropositive-infected bull with BLV and BHV-1.

^2^Seropositive-latently infected bull with BHV-1.

^3^Negative bull to BLV and BHV-1.

The assessment period seems to affect seminal quality and the percentage of bulls with doubtful seminal quality. During the initial evaluation, 70% (7/10) of the bulls showed at least one of the seminal parameters below the expected limit of detection for a sample with satisfactory seminal quality. At 30 days, only 40% (4/10) of the bulls showed doubtful seminal quality and the proportion of bulls with satisfactory seminal quality increased (Tables 1 and 2, Fig. 1).

**Figure 2:**
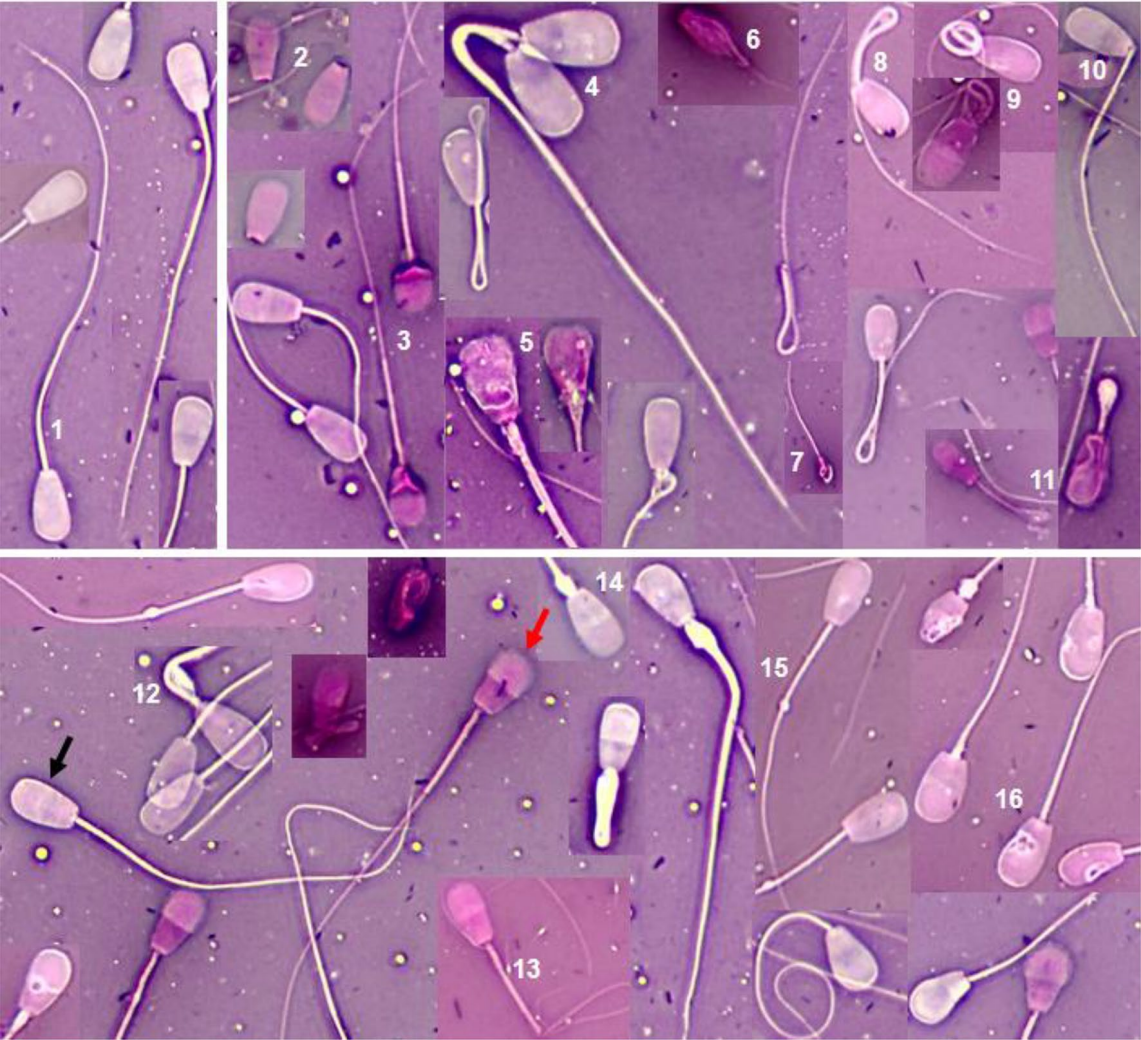
Abnormalities of bull spermatozoa stained with eosin-nigrosin. Sperm with normal cell membrane structure remained unstained (live, black arrow). Sperm with a damaged cell membrane stain pink (dead, red arrow). 1. Normal; 2. Loose head; 3. Severe pyriform head; 4. Double head; 5. Teratoid; 6. Inmature form; 7. Microcephaly; 8. Distal midpiece reflex; 9. Tightly coiled tail; 10. Broken neck; 11. Dag defect; 12. Bent midpiece; 13. Broken midpiece; 14. Proximal droplet; 15. Distal droplet; 16. Nuclear vacuoles based on Perry^31^.

Viability and percentage of abnormal sperm were the values with the best discriminant potential to determine the quality of fresh semen samples. The descriptive analysis of frequency of different types of sperm abnormalities at 30 days showed that all bulls had less than 30% abnormalities in the ejaculate. Four types of abnormalities had the highest prevalence: 100% (10/10) of bulls had sperm with proximal droplets, which ranged from 0.47 to 9.34%; 90% (9/10) of bulls had sperm with loose heads, which ranged from 0.47 to 18.93%; 90% (9/10) had sperm with tightly coiled tail, which ranged from 0.41 to 7.14% and, finally, 80% (8/10) had distal droplets, which ranged from 0.41 to 1.90%. There were also two rare anomalies in bulls, namely double heads and bent midpiece (Table 3, Fig. 2).

**Table 3:** Sanitary status and percentages of sperm anomalies in Brahman bulls (n = 10) at the end of the study (30 days).

Anomalies	Bull number^SS^									
	446^1^	498^1^	499^2^	518^2^	544^2^	545^2^	600^2^	616^2^	605^3^	606^3^
Loose head (%)	5.71	1.42	2.34	5.86	0.47	18.93	4.28	–	9.42	1.25
Pyriform head (%)	–	0.95	–	1.38	0.94	–	–	–	–	–
Double head (%)	–	–	–	––	–	–	–	–	–	0.41
Teratoid (%)	0.47	–	–	0.69	–	–	–	–	–	–
Inmature form (%)	–	0.95	–	1.38	1.81	0.82	–	–	0.82	–
Microcephaly (%)	–	0.47	–	0.69	0.94	2.05	–	–	0.82	0.41
Distal midpiece reflex (%)	1.90	4.76	–	3.10	3.69	–	–	5.71	2.46	0.41
Tightly coiled tail (%)	7.14	1.90	0.47	3.10	5.57	0.82	1.42	–	2.05	0.41
Dag defect (%)	–	–	–	–	–	–	–	–	2.05	–
Broken neck (%)						0.41				
Bent midpiece (%)	–	–	–	–	–	–	–	0.47	–	–
Broken midpiece (%)	–	–	–	0.34	–	–	–	–	–	–
Proximal droplet (%)	8.57	0.95	0.47	5.86	8.34	0.82	0.47	1.42	3.69	5.00
Distal droplet (%)	0.47	1.90	0.47	1.38	–	0.82	–	0.95	0.41	1.25
Nuclear vacuoles (%)	–	–	–	–	6.04	–	–	–	–	–
Total anomalies (%)	24.26	13.30	3.75	23.78	27.80	24.67	6.17	8.55	21.72	9.14

*SS* sanitary status: 1 = BLV^+^/BHV-1^+^, 2 = BLV^−^/BHV-1^+^, 3 = BLV^−^/BHV-1^−^.

The PFA rendered two principal components (PCs) for sperm kinematic variables, explaining 84.51% of the total variance (Table 4). The two PCs are similar in total variance explained. The PC1, named “linearity”, was positively correlated to progressivity parameters (LIN, STR and WOB). PC2, was named “velocity”, and was positively correlated to velocity parameters (VCL and VAP) and sperm head oscillation (ALH). The straight-line velocity (VSL) was included in the two PCs (Table 4).

**Table 4:** Eigenvectors of principal components (PCs)* for the sperm kinematic for bull.

Principal component*	PC1	PC2
LIN	0.99	
STR	0.80	
WOB	0.77	
VSL	0.75	0.62
VCL		0.99
ALH		0.89
VAP		0.87
Var Exp	43.03	41.48

*VCL* curvilinear velocity, *VSL* straight-line velocity. *VAP* average path velocity, *LIN* linearity of forward progression, *STR* straightness, *WOB* wobble, *ALH* amplitude of lateral head displacement.

*PC1* principal component designated “linearity”, *PC2* principal component designated “velocity”. *Var Exp* variance explained in each PC (%). Total variance explained = 84.51%.

*Expresses the more important variables in each PC. Only eigenvectors > 0.6 are presented.

Most of all kinematic parameters showed significant differences between different sanitary status. This means that the motility was more linear in a sanitary status BLV^−^/BHV-1^−^ than that of BLV^+^/BHV-1^+^ or BLV^−^/BHV-1^+^ bulls (Table 5). When comparing the sanitary status, VSL, VAP, LIN, STR, ALH and BCF were higher in BLV^−^/BHV-1^−^ than in BLV^+^/BHV-1^+^ and BLV^−^/BHV-1^+^ bulls. In contrast, WOB showed no differences among bulls with different sanitary status. When comparing VSL, VAP, LIN and STR, they were higher in BLV^−^/BHV-1^+^ that in BLV^+^/BHV-1^+^ bulls. Following these results, it seems that the sanitary status of a bull can affect the velocity and linearity of sperm kinematic parameters (Table 5).

**Table 5:** Descriptive statistics for the CASA-Mot variables (mean ± SEM) for each sanitary status for bulls.

Variable	Sanitary status		
	BLV^+^/BHV-1^+^	BLV^−^/BHV-1^+^	BLV^−^/BHV-1^−^
VCL	125.468 ± 0.420^a^	127.625 ± 0.266^b^	127.741 ± 0.443^b^
VSL	59.350 ± 0.283^a^	58.564 ± 0.179^b^	59.039 ± 0.299^c^
VAP	74.952 ± 0.283^a^	78.218 ± 0.179^b^	73.482 ± 0.299^c^
LIN	46.482 ± 0.189^a^	45.825 ± 0.120^b^	46.428 ± 0.199^c^
STR	75.768 ± 0.253^a^	72.665 ± 0.160^b^	76.798 ± 0.267^c^
WOB	59.398 ± 0.171^a^	60.563 ± 0.108^a^	58.551 ± 0.181^a^
ALH	4.169 ± 0.017^a^	4.191 ± 0.011^a^	4.256 ± 0.018^b^
BCF	9.959 ± 0.052^a^	9.785 ± 0.033^b^	10.614 ± 0.055^c^

*SEM* standard error of the mean. *BLV* bovine leukosis virus. *BHV-1* bovine herpesvirus 1.^+^Positive or ^−^Negative, presence. *VCL* (μm/s) curvilinear velocity, *VSL* (μm/s) straight-line velocity, *VAP* (μm/s) average path velocity, *LIN* (%) linearity of forward progression; *STR* (%) straightness; *WOB* (%) wobble, *ALH* (μm) amplitude of lateral head displacement, *BCF* (Hz): beat-cross frequency.

^a–c^Different superscripts within a row indicate significant differences; P < 0.05.

Four well-defined sperm kinematic subpopulations (SP1, SP2, SP3, SP4) were identified; they showed clear differences in most kinematic parameters (Table 6). SP1 had the lowest values in all kinematic parameters, being considered a "slow and nonlinear" subpopulation. SP2 had sperm with high values for VAP and VCL, but did not have high values for VSL, LIN and STR, and was regarded as "fast, but with less straight and linear" sperm than SP4. SP3 had high values for LIN, STR and WOB, but low values in VCL, VSL, VAP, being defined as “linear and slow” subpopulation. SP4 included spermatozoa with the highest linear trajectories (LIN, STR) and high speed (VSL, VAP) and BCF, and included fast straight, and linear sperm with a high tail beat (Table 6).

**Table 6:** Sperm kinematic variables (mean ± SEM) of the four ejaculate subpopulations.

Variable	Subpopulations (SPs)			
	1	2	3	4
VCL	64.163 ± 0.598^a^	182.085 ± 0.387^b^	85.310 ± 0.407^c^	176.219 ± 0.407^d^
VSL	17.264 ± 0.403^a^	54.057 ± 0.261^b^	52.215 ± 0.274^c^	112.402 ± 0.230^d^
VAP	30.085 ± 0.403^a^	94.754 ± 0.261^b^	58.566 ± 0.274^c^	118.797 ± 0.230^d^
LIN	28.048 ± 0.268^a^	29.905 ± 0.174^b^	62.176 ± 0.183^c^	64.850 ± 0.154^d^
STR	59.519 ± 0.359^a^	59.227 ± 0.233^a^	88.618 ± 0.244^b^	92.944 ± 0.205^c^
WOB	47.539 ± 0.244^a^	52.456 ± 0.158^b^	69.534 ± 0.166^c^	68.488 ± 0.139^d^
ALH	2.737 ± 0.024^a^	6.244 ± 0.015^b^	2.726 ± 0.016^a^	5.114 ± 0.014^c^
BCF	6.804 ± 0.074^a^	10.369 ± 0.048^b^	11.434 ± 0.051^c^	11.870 ± 0.043^d^

*SEM* standard error of the mean. (SPs) defined from bull semen samples. *VCL* (μm/s) curvilinear velocity, *VSL* (μm/s) straight-line velocity, *VAP* (μm/s) average path velocity, *LIN* (%) linearity of forward progression, *STR* (%) straightness, *WOB* (%) wobble, *ALH* (μm) amplitude of lateral head displacement, *BCF* (Hz): beat-cross frequency

^a–d^Different superscripts within a row indicate significant differences; P < 0.05.

The percentage of spermatozoa of each sanitary status in reference to the total number of spermatozoa varied between the subpopulations. The sanitary status BLV^−^/BHV-1^+^ (SS2) had the highest percentage of spermatozoa (54.3%), followed by BLV^−^/BHV-1^+^(SS1) with 23.1%, and finally by BLV^−^/BHV-1^−^ (SS3) with 22.7% of total spermatozoa analyzed. There was a higher percentage of SP4 in SS1 y SS2 (9.6% and 18.8%, respectively), but in SS3 was SP3 (7.6%) (Table 7). This means that bulls that were in SS1 y SS2 presented spermatozoa with high linear trajectories and speed.

**Table 7:** Percentage of each subpopulation (SP) in reference to the total number of spermatozoa the different sanitary status (SS).

SS		SP1	SP2	SP3	SP4
1	N	745^a^	1431^b^	1276^a^	2438^c^
	% within SS	12.6%	24.3%	21.7%	41.4%
	% within SP	21.5%	19.2%	22.5%	27.3%
	% of total	2.9%	5.6%	5.0%	9.6%
2	N	2187^a^	4393^b^	2467^c^	4798^d^
	% within SS	15.8%	31.7%	17.8%	34.7%
	% within SP	63.2%	59.1%	43.4%	53.7%
	% of total	8.6%	17.2%	9.7%	18.8%
3	N	531^a^	1613^b^	1940^c^	1706^d^
	% within SS	9.2%	27.9%	33.5%	29.5%
	% within SP	15.3%	21.7%	34.1%	19.1%
	% of total	2.1%	6.3%	7.6%	6.7%
Total	N	3463	7437	5683	8942
	% within SS	13.6%	29.1%	22.3%	35.0%

*n* number of spermatozoa. SS 1: BLV^+^/BHV-1^+^; SS 2: BLV^−^/BHV-1^+^; SS 3: BLV^−^/BHV-1^−^

^a–^^d^Different superscripts indicate significant differences; P < 0.05.

There were differences between sperm SPs within each SS (P < 0.05) with VCL and VAP exhibiting the highest differences between the sub-populations (Table 8). There were also differences between SPs for VSL and STR within SS2 and SS3 (P < 0.05), but not in SS1 (P > 0.05). For WOB there were differences between SPs only in SS2 and SS3; whereas for ALH there were differences between SPs only in SS1 and SS3. The SP distribution was not the same for each sanitary status (Table 8). When SP4 was compared between sanitary status, kinematics differences (P < 0.05) were found, for the variables VAP, LIN, STR and WOB. When SP1 was compared between sanitary status, kinematics differences (P < 0.05) were found only in WOB. The differences when comparing SP2 between the sanitary status presented a behavior similar to that shown in SP4, while the behavior of the differences in SP3 was similar to SP1. Following these results, SS1 and SS2 affect the kinematic variables of bull ejaculates.

**Table 8:** Sperm kinematics variables (mean ± SEM) of the four ejaculate subpopulations (SPs) in different sanitary status; defined from bull semen samples.

SPs	SP1	SP2	SP3	SP4
Sanitary status 1 (BLV^+^/BHV-1^+^)				
VCL	61.43 ± 1.082^aw^	176.768 ± 0.781^ax^	90.018 ± 0.827^ay^	173.654 ± 0.598^az^
VSL	16.572 ± 0.729^abw^	55.059 ± 0.526^ax^	54.662 ± 0.557^ax^	111.109 ± 0.403^ay^
VAP	28.372 ± 0.730^aw^	92.891 ± 0.526^ax^	61.752 ± 0.558^ay^	116.791 ± 0.403^az^
LIN	27.819 ± 0.486^aw^	31.396 ± 0.351^ax^	61.603 ± 0.371^ay^	65.112 ± 0.269^az^
STR	60.552 ± 0.651^aw^	61.114 ± 0.469^aw^	88.192 ± 0.497^ax^	93.216 ± 0.360^ay^
WOB	46.874 ± 0.441^aw^	52.871 ± 0.318^ax^	69.410 ± 0.337^ay^	68.439 ± 0.244^az^
ALH	2.691 ± 0.043^aw^	5.994 ± 0.031^ax^	2.961 ± 0.033^ay^	5.032 ± 0.024^az^
BCF	6.228 ± 0.135^aw^	10.594 ± 0.097^ax^	10.829 ± 0.103^ax^	12.185 ± 0.074^ay^
Sanitary status 2 (BLV ^−^/BHV-1^+^)				
VCL	62.168 ± 0.632^aw^	183.301 ± 0.446^bx^	87.445 ± 0.595^by^	177.587 ± 0.426^bz^
VSL	16.454 ± 0.426^aw^	48.754 ± 0.300^bx^	53.582 ± 0.401^ay^	115.465 ± 0.287^bz^
VAP	27.500 ± 0.426^aw^	99.661 ± 0.300^bx^	60.945 ± 0.401^ay^	124.767 ± 0.288^bz^
LIN	27.697 ± 0.284^aw^	26.813 ± 0.200^bw^	62.543 ± 0.267^bx^	66.245 ± 0.192^by^
STR	60.866 ± 0.380^aw^	51.170 ± 0.268^bx^	87.414 ± 0.358^ay^	91.212 ± 0.256^bz^
WOB	45.366 ± 0.258^bw^	54.677 ± 0.182^bx^	70.722 ± 0.242^by^	71.488 ± 0.174^bz^
ALH	2.763 ± 0.025^aw^	6.178 ± 0.018^bx^	2.789 ± 0.024^bw^	5.035 ± 0.017^ay^
BCF	6.225 ± 0.079^aw^	10.763 ± 0.055^ax^	10.865 ± 0.074^ax^	11.286 ± 0.053^by^
Sanitary status 3 (BLV^−^/BHV-1^−^)				
VCL	68.892 ± 1.282^bw^	186.187 ± 0.735^cx^	78.468 ± 0.671^cy^	177.416 ± 0.715^bz^
VSL	18.766 ± 0.864^bw^	58.358 ± 0.496^cx^	48.399 ± 0.452^by^	110.631 ± 0.482^az^
VAP	34.382 ± 0.864^bw^	91.711 ± 0.496^ax^	53.002 ± 0.452^by^	114.834 ± 0.482^cz^
LIN	28.629 ± 0.576^aw^	31.506 ± 0.330^ax^	62.383 ± 0.301^aby^	63.194 ± 0.321^cy^
STR	57.139 ± 0.771^bw^	65.398 ± 0.442^cx^	90.249 ± 0.403^by^	94.404 ± 0.430^cz^
WOB	50.377 ± 0.523^cw^	49.820 ± 0.300^cw^	68.471 ± 0.273^cx^	65.537 ± 0.292^cy^
ALH	2.758 ± 0.051^aw^	6.561 ± 0.029^cx^	2.428 ± 0.027^cy^	5.276 ± 0.029^bz^
BCF	7.958 ± 0.159^bw^	9.749 ± 0.091^bx^	12.609 ± 0.083^by^	12.140 ± 0.089^az^

*SEM* standard error of the mean. n: 25 525 spermatozoa analyzed. *VCL* (μm/s) curvilinear velocity, *VSL* (μm/s) straight-line velocity; VAP (μm/s) average path velocity, *LIN (%)* linearity, *STR (%)* straightness, *WOB (%)* wobble, *ALH* (μm) amplitude of lateral head displacement, *BCF* (Hz) beat-cross frequency.

^a–^^c^Values with different superscripts differ significantly between sanitary status (SS), P < 0.05.

^w–^^z^Values with different superscripts differ significantly between sperm subpopulations; P < 0.05.

## Discussion

The results of this study showed that the percentage of normal sperm morphology and sperm viability were the variables with the greatest discriminating potential for samples with satisfactory, doubtful, and unsatisfactory seminal quality. These results agree with those reported earlier^19–21^ but contrast with other studies which found that the percentage of abnormal spermatozoa was not a good predictor of fertility level^22^.

Our results indicate that bulls presented a spermiogram, which contained < 30% abnormal sperm, as a threshold value described in other studies^23–25^. However, there were three abnormal sperm morphology defects (loose head, tightly coiled tail and proximal droplet) that overcame 6% of individual thresholds for each abnormality^26^. The concept of sperm with compensable or uncompensable traits allows to differentiate thresholds as the case may be^27^. For example, compensable traits such as loose heads preclude affected sperm from fertilizing the ovum and the threshold in this case can be 30%^26^. For uncompensable traits the authors suggested that threshold of such abnormalities should not surpass 20%^26,28^. At 30 days, all the bulls showed a percentage of abnormal spermatozoa lower than 30%, a value associated with satisfactory quality for the seminal sample^29^. The prevalence of morphological abnormalities provides very valuable information to assess reproductive health associated with spermatogenesis^26^. When considering the prevalence of each sperm abnormality in each bull, the presence of proximal droplets was the most prevalent sperm abnormality, followed by loose heads, tightly coiled tails and distal droplets, which are within the 3 major types of sperm defects described for beef bulls^30^. The most serious morphological abnormalities were proximal droplets and loose heads. The high percentage of proximal droplets was 8.5% (Bull 446) but counts of 10–15% have been associated with decreased fertility^31^. Also, the highest percentage of detached heads was 18.9; although the bull can still be considered “fertile” with 30–40% of this defect, the ejaculate was considered infertile due to low viability (4%) (Bull 545). It has been described that the main causes of spermatozoa with detached heads are testicular hypoplasia, testicular degeneration and senescence of spermatozoa due to sexual inactivity. In this study the seminal samples of bulls were evaluated after the first ejaculation^30^. Our results were below threshold sperm values for compensable or uncompensable traits and provide evidence for the association between the sanitary status of seropositive-latently infected bull with BHV-1 and sperm morphology in Brahman bulls.

In general, it is accepted that bulls with values for progressive motility greater than 50% and normal morphology greater than 70% should be above the average fertility calculated for the sire group^29,30^. In this sense, the highest values for normal morphology, progressive motility and viability are positively associated with a higher non return rate than the average fertility in the group of bulls, but lower values of the seminal parameters expected as satisfactory for an ejaculate can be due to variability and do not indicate that the bull is subfertile. However, they do suggest that the bull with lower values should not be used for natural mating or as a semen donor in AI programs at the time^29,32^. Consequently, in this study high variability in sperm viability was observed between bulls with values that ranged from 4 to 93.8%. Particularly, in the group of bulls positive for BHV-1, it was observed that 50% (3/6) did not exceed 50% of viability, in accordance with what was reported by El-Mohamady et al.^33^ although it indicated that BHV-5, a virus similar to BHV-1, does not induce functional and morphological damage of infected spermatozoa. In this study the type of morphological abnormalities was very diverse and the percentage of abnormalities decreased in the second sample (14.4%; range 3.8–23.4%) compared to the first evaluation (35%; range 22.6–51.6%), which suggests an effect of the sampling period rather than the effect of the virus on semen quality, contrary to what was reported by El-Mohamady et al.^7^ because it was probably due to sperm turnover, in accordance to that indicated by the ABBA (2015). Recently, it has been reported in Brahman bulls that sexual rest decreases sperm quality^34^. More research is necessary on the issue of dead sperm in inactive/rested bulls and exploring the possibility that sperm quality is better in the second sample.

In reference to the CASA variables, it seems that they do not contribute to differentiate seminal quality with a discriminant effect^35^. The differences observed in values of semen parameters for each bull, considering the sanitary status and the evaluations (0 and 30 days), show considerable variability and no clear associations were observed^2^. Furthermore, some authors suggested that BHV-1 does not affect semen quality^33,36^. Our findings indicate that although there were differences between sanitary status of Brahman bulls in kinematics variables, this did not appear to have a biological relationship with velocity, linearity, or undulation of sperm cells.

Based on CASA-Mot variables, our results identified four sperm subpopulations in Brahman bulls. The differences between sperm subpopulations (P < 0.05) suggest that sperm kinematics patterns with very active but non-progressive movement or rapid and progressive sperm movement may be indicative of early capacitation and hyperactivated-like motile spermatozoa. Other sperm subpopulations contained poorly motile sperm, and others presented non-progressive sperm and low velocity but greater progressive kinematics traits which highlights the heterogeneous nature of sperm ejaculates of fresh semen in Brahman bulls^18,37^. The analysis of sperm subpopulations by sanitary status provides evidence that the pattern of high velocity and progressive motility is associated with BLV^−^/BHV-1^+^ and BLV^−^/BHV-1^−^, this suggestes that seropositive-infected bulls with BLV and BHV-1 should be discarded as sires.

The evaluation of seminal quality is very complex. Apparently, the variables that best discriminate between the quality of the ejaculates are normal morphology and sperm viability. The CASA-Mot variables do not reveal a clear trend with regards to the quality of semen samples. Taken together, the sanitary status of bulls did not show a clear change in the trend of the variables that define semen quality. However, the results indicated that the bulls infected with BLV or BHV-1 produce semen with variable quality, and that in most of the bulls the semen was free of the presence of the virus, but the use of bulls infected with BLV can generate semen contaminated in an intermittent way. The sperm subpopulation analysis indicated that seropositive-infected bulls with BLV and BHV-1 should be excluded from beef cattle farms.

## Materials and methods

This study was performed following ethical principles and with the approval of the Committee of Centro de Investigación y Desarrollo de la Agricultura Sostenible para el Trópico Húmedo at the Costa Rica Institute of Technology (CIDASTH-ITCR) according to section 01/2019, article 1.0, DAGSC-074-2019. The care of the animals during the research complied with the animal welfare guidelines of Costa Rica Institute of Technology. Bulls were grazed and sampling procedures were reviewed and approved by the Production Committee of the Agricultural Production Program of the School of Agronomy at the Costa Rica Institute of Technology (PPASC-106-2021). A statement about plant ethics is not relevant because no experimentation is done on plants. The study was carried out in compliance with ARRIVE guidelines (https://arriveguidelines.org/; accessed on 8 November 2021).

### Animals

Ten randomly, apparently healthy, Brahman bulls in sexual rest (≈ 3 months) were employed in this study. The animals came from a commercial farm located in San Carlos, Costa Rica. The bulls had passed a standard breeding soundness evaluation and had produced sperm with acceptable post-thaw characteristics (progressive motile sperm > 60%) and fertility (non-return rates > 65%).

The sanitary status of bulls against BLV and BHV-1 was previously established by enzyme-linked immunosorbent assay (ELISA) [BLV^+^/BHV-1^+^, (n = 2); BLV^−^/BHV-1^+^, (n = 6) and BLV^−^/BHV-1^−^, (n = 2)]. The age of the animals ranged from 25 to 84 months, body weight ranged from 400 to 850 kg, mean scrotal circumference was 37.6 ± 2.9 cm, and mean scrotal temperature was 32.1 ± 0.5 °C. Bulls included in the study were negative for the presence of antibodies and antigens associated with circulating bovine viral diarrhea virus. On the farm, the animals were not vaccinated against infectious bovine rhinotracheitis.

### Animal handling

During the experimental period, the animals were handled in an area of 18 hectares, a rotational system of seven apartments was established, with six days of occupation and 42 days of rest, with Ratana grass (*Ischaemum indicum*) and Mombaza (*Panicum maximum*). Animals were fed with grass without restrictions of access to forage consumption and they were supplied with salt, minerals and water ad libitum. The sanitary management of the bulls were an external dewormer (e.g., Doramectin), water-soluble vitamins (Catosal®) and minerals (Matsuda breeding Top Line). General inspection of the bulls was made, focusing on anatomical aspects such as conformation of legs and feet, body condition, coloration of mucous membranes and particularities of each animal. The condition of the external reproductive organs, such as the testicles, scrotum, foreskin, and penis were also examined.

### Semen collection and processing

Semen was collected by electroejaculation using the Pulsator V® equipment (Lane Manufacturing Inc, Denver, CO, USA) as described by Montoya-Monsalve et al., and Víquez et al.^2,37^, under a collection program of paired semen samples. Semen was obtained twice, 30 days apart, early in the morning from ten bulls in sexual rest. Semen was collected directly from the penis using a sterile graduate collection tube to assess the volume, each ejaculate was diluted at a ratio of 1:1 (vol/vol) using a commercial egg yolk-free diluent (OptiXcell 2-IMV Technologies, L’Aigle, France). Prior to semen dilution, a 1 mL aliquot of raw semen was placed in 1.5 mL sterile microtubes. Semen samples were assessed for gross motility, by placing 20 µL of fresh raw semen on a prewarmed slide at 37 °C, and for concentration, employing a bovine photometer AccuRead® (IMV Technologies, L’Aigle, France) at 530 nm wavelength. Diluted semen samples were placed on a heating plate at 37 °C and then transported in a polyurethane cooler at 25 °C. Subsequently, the raw semen samples were frozen at − 20 °C until nucleic acid extraction.

Viability, percentage of sperm abnormalities and sperm morphology were assessed by preparing a smear of the semen sample using 10 µL of diluted semen stained with eosin-nigrosin as described by Kumar et al.^38^ and at least 200 spermatozoa were counted to determine the percentage of abnormalities^22^. All samples were coded in such a way that the technician who performed the analysis did not know the number of the bull, the number of the ejaculate, or which ejaculate belonged to a particular bull.

After semen extraction, blood samples with and without anticoagulant were taken by coccygeal vein puncture using the Vacutainer system, and placed in a cooler at 4 °C. An aliquot of 1.5 mL of blood with anticoagulant was deposited in 1.5 mL microtubes and frozen at − 20 °C until the extraction of nucleic acids. Serum was separated by centrifugation at 3500×*g* 5 min, an aliquot of 1.5 mL was taken and stored in 1.5 mL microtubes at − 20 °C until assessment.

### Semen analysis

#### Assessment of sperm variables

Reusable counting chambers with cover slide for the analysis of motility and kinematics variables (Spermtrack® 20-micron depth, Proiser R + D, S.L., Paterna, Spain) were used after pre-warming them to 37 °C^39^. After thorough mixing of the diluted semen samples, 2.7 µL of diluted semen were placed in the counting chamber tracks by drop displacement according to Valverde et al.^40^. Analyses were conducted with the CASA-Mot system ISAS®v1 (Integrated Semen Analysis System, Proiser R + D, Paterna, Spain) fitted with a video-camera (Proiser 782 M, Proiser R + D), a frame rate of 50 frames per second (fps) and a final resolution of 768 × 576 pixels as described by Valverde et al^41^. The camera was attached to a microscope UB203 (UOP/Proiser R + D) with a 1X eyepiece and a 10X negative-phase contrast objective (AN 0.25), and an integrated heated stage maintained at 37 ± 0.5 °C.

#### Computerized kinematic analysis

CASA analyses were performed recording seven microscope fields with a total of at least 600 cells per sample. The CASA-Mot variables assessed in this study included: straight-line velocity (VSL, µm·s^−1^), corresponding to the straight line from the beginning to the end of the track; curvilinear velocity (VCL, µm/s), measured over the actual point-to-point track followed by the cell; average path velocity (VAP, µm/s), the average velocity over the smoothed cell path; amplitude of lateral head displacement (ALH, µm), defined as the maximum of the measured width of the head oscillation as the sperm swims; beat-cross frequency (BCF, Hz), defined as the frequency with which the actual track crosses the smoothed track in either direction; motility (%), the percentage of total motile cells and progressive motility (%), corresponding to spermatozoa swimming rapidly forward in a straight line (assessed as straightness index ≥ 45%; VAP ≥ 25 µm/s). Three progression ratios, expressed as percentages, were calculated from the velocity measurements described above: linearity of forward progression (LIN = VSL/VCL·100), straightness (STR = VSL/VAP·100), and wobble (WOB = VAP/VCL·100)^17^.

### Sanitary status for BLV and BHV-1

#### Serologic assessment using enzyme-linked immunosorbent assays

The sanitary status of the bulls for BLV and BHV-1 was determined in the Veterinary Virology Diagnostic and Research Unit, School of Veterinary Medicine, National University, Costa Rica using ID Screen® BLV gp51 Competition and ID Screen® IBR gB Competition (ID.vet, Grabels, France) according to the manufacturer's instructions, respectively.

#### Antigenic titration by polymerase chain reaction

Genomic DNA extraction was performed from 300 µL of blood with EDTA and from semen diluted 1:5 (vol:vol) using Wizard® Genomic DNA Purification Kit (Promega Corporation, Madison, USA) according to the manufacturer’s instructions for DNA extraction from samples of frozen blood. The DNA obtained was stored at -75 °C until use. BLV proviral DNA was identified by semi-nested PCR. In the first round, a set of primer targeting on gene encoding gp51 (envelope protein) were used, the forward primer was Env1: 5′-ATGCCYAARRAACGACGRYCCCG-3′ (4826–4848), and the reverse primer was Env3: 5′-GAGGGCGAGRCCGGGTCCAGAGC-3′ (5550–5528), while the second round was performed with the forward primer Env2: 5′-CAAGGGCGRCGCCRRTTYGGAGC-3′ (5108–5124) and the reverse primer Env3 described above. The final volume of each PCR reaction was 20 µL and the mix consisted of 10 µL of Phusion U Multiplex PCR Master Mix #F-562S (Invitrogen, USA); 1.2 μL (0.3 μM/μL) of each primer pair Env1(+)/Env3(−) in the first round and Env2(+)/Env3(−) in the second round, 6.6 μL of RNase-free water (Invitrogen, USA) and 1 μL of proviral DNA in the first reaction or 1 μL of the initial PCR mixture of the first reaction after amplification in the second round to complete the volume for nested PCR. The amplification protocol was the same in both PCRs, as follows: initial denaturation at 98 °C for 3 min, 35 cycles of denaturation at 98 °C for 20 s, annealing at 70 °C for 30 s, and extension at 72 °C for 15 s, followed by a final extension at 72 °C during 5 min and maintenance at 4 °C thereafter. DNA from strain 280918-4418-CR isolated in Costa Rica was used as positive control (GenBank accession number: MN830810), while Rnase free water (Invitrogen, USA) was used as negative control. The expected products for BLV were 725 and 443 bp when the Env1(+)/Env3(−) primer pair was used in the first round or when the Env2(+)/Env3(−) primer pair was used in the second round, respectively, according to Khudhair et al^42^.

BHV-1 was identified using a set of primers that were based on region of the gE gen of BHV-1. The forward primer was BHV1-gE3-883F: 5′-CAATAACAGCGTAGACCTGGTC-3′ (122,739–122,760), and the reverse primer was BHV1-gE3-989R: 5′-GCTGTAGTCCCAAGCTTCCAC-3′ (122,844–122,824) (Wernike et al., 2011). The final volume of each PCR reaction was 20 µL and consisted of 10 µL of Phusion U Multiplex PCR Master Mix #F562S (Invitrogen, USA), 1 µL of each primer IBR-IgE-F(+)/IBR-IgE(−) (0.25 µM/µL), 7 µL of Rnase-free H2O (Invitrogen, USA) and 1 µL of DNA. The amplification protocol consisted of an initial denaturation cycle at 98 °C for 2 min; 35 cycles of denaturation at 98 °C for 20 s, annealing at 63 °C for 30 s and amplification at 72 °C for 15 s, followed by a final amplification at 72 °C during 5 min and holding at 4 °C thereafter. DNA from the Colorado strain contained in the commercial vaccine EXPRESS® FP 10-HS (Boehringer Ingelheim Vetmedica, Germany) was used as a positive control. The expected product was 106 bp. All reactions were carried out in the 2720 thermocycler (Applied Biosystems, USA). The products amplified by PCR were stained with GelRed Nucleic Acid Stain 10000X in Water (Biotium, USA) and separated on a 2% agarose gel using the Fotodyne Model N°1-1430 electrophoresis equipment at 80 V during 40 min. Once separated they were visualized in the imager and ultraviolet illuminator (UVP BioDoc-1tTM Imaging System/FirstLight UV illuminator, USA).

### Statistical analysis

A normal probability plot was used to assess normal distribution. The data obtained for the analysis of all sperm variables were assessed for homoscedasticity by using Levene test. Further, sperm variables were analyzed using the Generalized Linear Mixed Models (GLMM). The response variables were semen volume, total and progressive motility, swimming patterns (kinematics of spermatozoa), sperm concentration, normal and abnormal sperm (%), and viability (%). A normal distribution with an identity link function was assumed for all response variables. The quality of the seminal samples was categorized by analyzing the percentage of three variables (progressive motility, normal morphology, and viability when information was available), as follows: satisfactory seminal quality (*S* = total progressive motility > 50%, normal morphology > 70% and viability > 50%), doubtful (*D* = if at least one of the three variables did not reach satisfactory values) and unsatisfactory (*U* = if three or at least two of the variables did not reach satisfactory levels^19,20,29^. The association between semen quality with the sanitary status was evaluated descriptively.

#### Multivariate procedures

A subset of data was created with the means per ejaculate of all eight morphometric variables. Multivariate procedures were performed to identify sperm clusters from this subset of sperm morphometric data. All the values for the morphometric variables were standardized to avoid any scale effect. A principal factor analysis (PFA) was performed on these data to derive a small number of linear combinations that still retained as much information as possible from the original variables. Prior communalities for this analysis were estimated from the maximum absolute correlation coefficient between each variable and any other. The number of principal factors (PF) to be extracted was determined from the Kaiser criterion, namely by selecting only those with an eigenvalue > 1. The KMO (Kaiser–Meyer–Olkin) statistic was also obtained^43^ as a measure of dataset adequacy for factor extraction. As a rotation method, the varimax method with Kaiser normalization was used^44^.

Further, an analysis was conducted to classify the ejaculates into a reduced number of clusters, based on scores obtained from factor analysis. This was accomplished in two-step phases, combining hierarchical and non-hierarchical clustering procedures with the sperm-derived indices obtained after the PFA. First, factor scores for all ejaculates were clustered hierarchically using the Ward Minimum Variance method^45^. From this analysis, an optimal number of clusters was determined based on criteria such as the Cubic Clustering Criterion (CCC), Pseudo-T, Pseudo-F, and partial R2. Second, the optimal number of clusters obtained in the previous analysis was used as the target number of clusters in a non-hierarchical K-means model cluster analysis^46^.

This classifies the spermatozoa of the data set into a small number of cluster (subpopulations) according to their head dimensions or kinematics variables, as has been described previously by Barquero et al.^47^. This analysis allowed the identification of sperm subpopulation and the detection of outliers. Analysis of variance (ANOVA) was further applied to evaluate statistical differences between clusters for all morphometric variables. Furthermore, an interaction effect between sanitary status and sperm subpopulation was considered. The threshold for significance was defined as P < 0.05. Multiple comparisons between cluster means were performed by the Tukey adjustment. Results were presented as mean ± standard error of the mean. All data were analyzed using the IBM SPSS statistical program, version 23.0 for Windows (SPSS Inc., Chicago, IL, USA).

### Institutional review board statement

The study was conducted following ethical principles and the approval of the Committee of Centro de Investigación y Desarrollo de la Agricultura Sostenible para el Trópico Húmedo at the Costa Rica Institute of Technology (CIDASTH-ITCR) according to Section 01/2019, article 1.0, DAGSC-074-2019, and Production Committee of the Agricultural Production Program of the School of Agronomy at the Costa Rica Institute of Technology (PPASC-106-2021).

## Data Availability

The data presented in this study are available within the article.
